
JOURNAL INFORMATION
==============================
NLM Title Abbreviation: Sci Pharm
Journal ID: Sci Pharm
NLM Journal ID: 0026251
Journal ID: Scientia Pharmaceutica

Scientia Pharmaceutica

ISSN: 0036-8709
EISSN: 2218-0532
Publisher: Österreichische Apotheker-Verlagsgesellschaft, m. b. H.

ARTICLE INFORMATION
==============================
PMCID: PMC3002806
Article ID: scipharm.2010.78.541
Article version: 1
Subjects: Abstracts of the 8th Central European Symposium on Pharmaceutical Technology (CESPT), Satellite Symposium: 4th International Graz Congress on Pharmaceutical Engineering, September 16th-18th, Graz, Austria

Keynote Lectures (L)

Electronic publication date: 2010 Sep 30
Print publication date: 2010 Jul-Sep
Volume: 78
Issue: 3
First page: 541
Last page: 554
Copyright: © author(s); licensee Österreichische Apotheker-Verlagsgesellschaft m. b. H., Vienna, Austria.
Copyright year: 2010
License: This is an Open Access article distributed under the terms of the Creative Commons Attribution License (http://creativecommons.org/licenses/by/3.0/), which permits unrestricted use, distribution, and reproduction in any medium, provided the original work is properly cited.
License URL: https://creativecommons.org/licenses/by/3.0/

==============================
JOURNAL INFORMATION
==============================
NLM Title Abbreviation: Sci Pharm
Journal ID: Sci Pharm
NLM Journal ID: 0026251
Journal ID: Scientia Pharmaceutica

Scientia Pharmaceutica

ISSN: 0036-8709
EISSN: 2218-0532
Publisher: Österreichische Apotheker-Verlagsgesellschaft, m. b. H.

ARTICLE INFORMATION
==============================
DOI: 10.3797/scipharm.cespt.8.L01
Article ID: scipharm.2010.78.541a
Subjects: Conference abstract L01

Drug Delivery in the GI Tract: How Controlled is Controlled Release?

Weitschies W.
Department of Biopharmaceutics and Pharmaceutical Technology, Institute of Pharmacy, University of Greifswald, Germany
E-mail: werner.weitschies@uni-greifswald.de
Electronic publication date: 2010 Sep 30
Print publication date: 2010 Jul-Sep
Volume: 78
Issue: 3
First page: 541
Last page: 541
Copyright: © author(s); licensee Österreichische Apotheker-Verlagsgesellschaft m. b. H., Vienna, Austria.
Copyright year: 2010
License: This is an Open Access article distributed under the terms of the Creative Commons Attribution License (http://creativecommons.org/licenses/by/3.0/), which permits unrestricted use, distribution, and reproduction in any medium, provided the original work is properly cited.

JOURNAL INFORMATION
==============================
NLM Title Abbreviation: Sci Pharm
Journal ID: Sci Pharm
NLM Journal ID: 0026251
Journal ID: Scientia Pharmaceutica

Scientia Pharmaceutica

ISSN: 0036-8709
EISSN: 2218-0532
Publisher: Österreichische Apotheker-Verlagsgesellschaft, m. b. H.

ARTICLE INFORMATION
==============================
DOI: 10.3797/scipharm.cespt.8.L02
Article ID: scipharm.2010.78.542
Subjects: Conference abstract L02

Nanofibers, nanofluidics, nanoparticles and nanobots for drug and protein delivery systems

Yarin A. L.
University of Illinois at Chicago, USA
E-mail: ayarin@uic.edu
Electronic publication date: 2010 Sep 30
Print publication date: 2010 Jul-Sep
Volume: 78
Issue: 3
First page: 542
Last page: 542
Copyright: © author(s); licensee Österreichische Apotheker-Verlagsgesellschaft m. b. H., Vienna, Austria.
Copyright year: 2010
License: This is an Open Access article distributed under the terms of the Creative Commons Attribution License (http://creativecommons.org/licenses/by/3.0/), which permits unrestricted use, distribution, and reproduction in any medium, provided the original work is properly cited.

JOURNAL INFORMATION
==============================
NLM Title Abbreviation: Sci Pharm
Journal ID: Sci Pharm
NLM Journal ID: 0026251
Journal ID: Scientia Pharmaceutica

Scientia Pharmaceutica

ISSN: 0036-8709
EISSN: 2218-0532
Publisher: Österreichische Apotheker-Verlagsgesellschaft, m. b. H.

ARTICLE INFORMATION
==============================
DOI: 10.3797/scipharm.cespt.8.L03
Article ID: scipharm.2010.78.543
Subjects: Conference abstract L03

Continuous Pharmaceutical Manufacture Using a Novel Wet Granulation Technique

Seville J. P. K.
Waters M.
School of Engineering, University of Warwick, Coventry, England
E-mail: J.P.K.Seville@warwick.ac.uk (J. P. K. Seville)
Electronic publication date: 2010 Sep 30
Print publication date: 2010 Jul-Sep
Volume: 78
Issue: 3
First page: 543
Last page: 543
Copyright: © author(s); licensee Österreichische Apotheker-Verlagsgesellschaft m. b. H., Vienna, Austria.
Copyright year: 2010
License: This is an Open Access article distributed under the terms of the Creative Commons Attribution License (http://creativecommons.org/licenses/by/3.0/), which permits unrestricted use, distribution, and reproduction in any medium, provided the original work is properly cited.

JOURNAL INFORMATION
==============================
NLM Title Abbreviation: Sci Pharm
Journal ID: Sci Pharm
NLM Journal ID: 0026251
Journal ID: Scientia Pharmaceutica

Scientia Pharmaceutica

ISSN: 0036-8709
EISSN: 2218-0532
Publisher: Österreichische Apotheker-Verlagsgesellschaft, m. b. H.

ARTICLE INFORMATION
==============================
DOI: 10.3797/scipharm.cespt.8.L04
Article ID: scipharm.2010.78.544
Subjects: Conference abstract L04

Chemical Swarm Robots: Design, Synthesis and Functionality

Stepanek F.
Chemical Robotics Laboratory, Institute of Chemical Technology, Prague, Czech Republic
E-mail: Frantisek.Stepanek@vscht.cz
Electronic publication date: 2010 Sep 30
Print publication date: 2010 Jul-Sep
Volume: 78
Issue: 3
First page: 544
Last page: 544
Copyright: © author(s); licensee Österreichische Apotheker-Verlagsgesellschaft m. b. H., Vienna, Austria.
Copyright year: 2010
License: This is an Open Access article distributed under the terms of the Creative Commons Attribution License (http://creativecommons.org/licenses/by/3.0/), which permits unrestricted use, distribution, and reproduction in any medium, provided the original work is properly cited.

JOURNAL INFORMATION
==============================
NLM Title Abbreviation: Sci Pharm
Journal ID: Sci Pharm
NLM Journal ID: 0026251
Journal ID: Scientia Pharmaceutica

Scientia Pharmaceutica

ISSN: 0036-8709
EISSN: 2218-0532
Publisher: Österreichische Apotheker-Verlagsgesellschaft, m. b. H.

ARTICLE INFORMATION
==============================
DOI: 10.3797/scipharm.cespt.8.L05
Article ID: scipharm.2010.78.545
Subjects: Conference abstract L05

Nanomedicine for Drug Delivery across Epithelial Barriers: Intestines, Skin and Lungs

Lehr C.-M.
Helmholtz Institute of Pharmaceutical Research, Saarland University, Saarbrücken Germany
E-mail: lehr@mx.uni-saarland.de
Electronic publication date: 2010 Sep 30
Print publication date: 2010 Jul-Sep
Volume: 78
Issue: 3
First page: 545
Last page: 545
Copyright: © author(s); licensee Österreichische Apotheker-Verlagsgesellschaft m. b. H., Vienna, Austria.
Copyright year: 2010
License: This is an Open Access article distributed under the terms of the Creative Commons Attribution License (http://creativecommons.org/licenses/by/3.0/), which permits unrestricted use, distribution, and reproduction in any medium, provided the original work is properly cited.

JOURNAL INFORMATION
==============================
NLM Title Abbreviation: Sci Pharm
Journal ID: Sci Pharm
NLM Journal ID: 0026251
Journal ID: Scientia Pharmaceutica

Scientia Pharmaceutica

ISSN: 0036-8709
EISSN: 2218-0532
Publisher: Österreichische Apotheker-Verlagsgesellschaft, m. b. H.

ARTICLE INFORMATION
==============================
DOI: 10.3797/scipharm.cespt.8.L06
Article ID: scipharm.2010.78.546
Subjects: Conference abstract L06

Nanoparticles for Cell Specific Drug Delivery

Langer K.
Institut für Pharmazeutische Technologie und Biopharmazie, Westfälische Wilhelms-Universität, Münster, Germany
E-mail: k.langer@uni-muenster.de
Electronic publication date: 2010 Sep 30
Print publication date: 2010 Jul-Sep
Volume: 78
Issue: 3
First page: 546
Last page: 546
Copyright: © author(s); licensee Österreichische Apotheker-Verlagsgesellschaft m. b. H., Vienna, Austria.
Copyright year: 2010
License: This is an Open Access article distributed under the terms of the Creative Commons Attribution License (http://creativecommons.org/licenses/by/3.0/), which permits unrestricted use, distribution, and reproduction in any medium, provided the original work is properly cited.

JOURNAL INFORMATION
==============================
NLM Title Abbreviation: Sci Pharm
Journal ID: Sci Pharm
NLM Journal ID: 0026251
Journal ID: Scientia Pharmaceutica

Scientia Pharmaceutica

ISSN: 0036-8709
EISSN: 2218-0532
Publisher: Österreichische Apotheker-Verlagsgesellschaft, m. b. H.

ARTICLE INFORMATION
==============================
DOI: 10.3797/scipharm.cespt.8.L07
Article ID: scipharm.2010.78.547
Subjects: Conference abstract L07

Toxicokinetics of Insoluble Nanoparticles in Rodents after Different Routes of Administration

Kreyling W. G.
Helmholtz Center Munich – Research Center for Environmental Health; Comprehensive Pneumology Center – Institute for Lung Biology and Disease and Focus Network NanoHealth, 85764 Neuherberg / Munich, Germany
E-mail: kreyling@helmholtz-muenchen.de
Electronic publication date: 2010 Sep 30
Print publication date: 2010 Jul-Sep
Volume: 78
Issue: 3
First page: 547
Last page: 547
Copyright: © author(s); licensee Österreichische Apotheker-Verlagsgesellschaft m. b. H., Vienna, Austria.
Copyright year: 2010
License: This is an Open Access article distributed under the terms of the Creative Commons Attribution License (http://creativecommons.org/licenses/by/3.0/), which permits unrestricted use, distribution, and reproduction in any medium, provided the original work is properly cited.

JOURNAL INFORMATION
==============================
NLM Title Abbreviation: Sci Pharm
Journal ID: Sci Pharm
NLM Journal ID: 0026251
Journal ID: Scientia Pharmaceutica

Scientia Pharmaceutica

ISSN: 0036-8709
EISSN: 2218-0532
Publisher: Österreichische Apotheker-Verlagsgesellschaft, m. b. H.

ARTICLE INFORMATION
==============================
DOI: 10.3797/scipharm.cespt.8.L08
Article ID: scipharm.2010.78.548
Subjects: Conference abstract L08

A 3D model of the epithelial airway barrier to study uptake, cell responses and intracellular distribution of nanoparticles

Rothen-Rutishauser B.
Brandenberger C.
Lehmann A.
Gehr P.
Institute of Anatomy, University of Bern, Bern, Switzerland
E-mail: rothen@ana.unibe.ch (B. Rothen-Rutishauser)
Electronic publication date: 2010 Sep 30
Print publication date: 2010 Jul-Sep
Volume: 78
Issue: 3
First page: 548
Last page: 548
Copyright: © author(s); licensee Österreichische Apotheker-Verlagsgesellschaft m. b. H., Vienna, Austria.
Copyright year: 2010
License: This is an Open Access article distributed under the terms of the Creative Commons Attribution License (http://creativecommons.org/licenses/by/3.0/), which permits unrestricted use, distribution, and reproduction in any medium, provided the original work is properly cited.

JOURNAL INFORMATION
==============================
NLM Title Abbreviation: Sci Pharm
Journal ID: Sci Pharm
NLM Journal ID: 0026251
Journal ID: Scientia Pharmaceutica

Scientia Pharmaceutica

ISSN: 0036-8709
EISSN: 2218-0532
Publisher: Österreichische Apotheker-Verlagsgesellschaft, m. b. H.

ARTICLE INFORMATION
==============================
DOI: 10.3797/scipharm.cespt.8.L09
Article ID: scipharm.2010.78.549
Subjects: Conference abstract L09

Process Analytical Technology Enabling Continuous Drug Product Manufacturing

Hammond S.
Moshgbar M.
Pfizer Inc, Morris Plains, New Jersey, USA
E-mail: steve.hammond@pfizer.com (S. Hammond)
Electronic publication date: 2010 Sep 30
Print publication date: 2010 Jul-Sep
Volume: 78
Issue: 3
First page: 549
Last page: 549
Copyright: © author(s); licensee Österreichische Apotheker-Verlagsgesellschaft m. b. H., Vienna, Austria.
Copyright year: 2010
License: This is an Open Access article distributed under the terms of the Creative Commons Attribution License (http://creativecommons.org/licenses/by/3.0/), which permits unrestricted use, distribution, and reproduction in any medium, provided the original work is properly cited.

JOURNAL INFORMATION
==============================
NLM Title Abbreviation: Sci Pharm
Journal ID: Sci Pharm
NLM Journal ID: 0026251
Journal ID: Scientia Pharmaceutica

Scientia Pharmaceutica

ISSN: 0036-8709
EISSN: 2218-0532
Publisher: Österreichische Apotheker-Verlagsgesellschaft, m. b. H.

ARTICLE INFORMATION
==============================
DOI: 10.3797/scipharm.cespt.8.L10
Article ID: scipharm.2010.78.550
Subjects: Conference abstract L10

Spectroscopic Investigations of Drug Substances and Drug Formulations

Rades T.
Chair in Pharmaceutical Sciences, The New Zealand National School of Pharmacy, PO Box 56 18 Frederick Street, Adams Building, Dunedin, New Zealand
E-mail: thomas.rades@otago.ac.nz
Electronic publication date: 2010 Sep 30
Print publication date: 2010 Jul-Sep
Volume: 78
Issue: 3
First page: 550
Last page: 550
Copyright: © author(s); licensee Österreichische Apotheker-Verlagsgesellschaft m. b. H., Vienna, Austria.
Copyright year: 2010
License: This is an Open Access article distributed under the terms of the Creative Commons Attribution License (http://creativecommons.org/licenses/by/3.0/), which permits unrestricted use, distribution, and reproduction in any medium, provided the original work is properly cited.

JOURNAL INFORMATION
==============================
NLM Title Abbreviation: Sci Pharm
Journal ID: Sci Pharm
NLM Journal ID: 0026251
Journal ID: Scientia Pharmaceutica

Scientia Pharmaceutica

ISSN: 0036-8709
EISSN: 2218-0532
Publisher: Österreichische Apotheker-Verlagsgesellschaft, m. b. H.

ARTICLE INFORMATION
==============================
DOI: 10.3797/scipharm.cespt.8.L11
Article ID: scipharm.2010.78.551
Subjects: Conference abstract L11

What do we Know about Tablet Coating Uniformity?

Kleinebudde P.
Institute of Pharmaceutics and Biopharmaceutics, Heinrich-Heine-University, Düsseldorf, Germany
E-mail: kleinebudde@uni-duesseldorf.de
Electronic publication date: 2010 Sep 30
Print publication date: 2010 Jul-Sep
Volume: 78
Issue: 3
First page: 551
Last page: 551
Copyright: © author(s); licensee Österreichische Apotheker-Verlagsgesellschaft m. b. H., Vienna, Austria.
Copyright year: 2010
License: This is an Open Access article distributed under the terms of the Creative Commons Attribution License (http://creativecommons.org/licenses/by/3.0/), which permits unrestricted use, distribution, and reproduction in any medium, provided the original work is properly cited.

JOURNAL INFORMATION
==============================
NLM Title Abbreviation: Sci Pharm
Journal ID: Sci Pharm
NLM Journal ID: 0026251
Journal ID: Scientia Pharmaceutica

Scientia Pharmaceutica

ISSN: 0036-8709
EISSN: 2218-0532
Publisher: Österreichische Apotheker-Verlagsgesellschaft, m. b. H.

ARTICLE INFORMATION
==============================
DOI: 10.3797/scipharm.cespt.8.L12
Article ID: scipharm.2010.78.552
Subjects: Conference abstract L12

Melt Granulation as a Modern Technological Procedure

Regdon G. Jr.
Department of Pharmaceutical Technology, University of Szeged, Szeged, Hungary
E-mail: geza.regdon@pharm.u-szeged.hu
Electronic publication date: 2010 Sep 30
Print publication date: 2010 Jul-Sep
Volume: 78
Issue: 3
First page: 552
Last page: 552
Copyright: © author(s); licensee Österreichische Apotheker-Verlagsgesellschaft m. b. H., Vienna, Austria.
Copyright year: 2010
License: This is an Open Access article distributed under the terms of the Creative Commons Attribution License (http://creativecommons.org/licenses/by/3.0/), which permits unrestricted use, distribution, and reproduction in any medium, provided the original work is properly cited.

JOURNAL INFORMATION
==============================
NLM Title Abbreviation: Sci Pharm
Journal ID: Sci Pharm
NLM Journal ID: 0026251
Journal ID: Scientia Pharmaceutica

Scientia Pharmaceutica

ISSN: 0036-8709
EISSN: 2218-0532
Publisher: Österreichische Apotheker-Verlagsgesellschaft, m. b. H.

ARTICLE INFORMATION
==============================
DOI: 10.3797/scipharm.cespt.8.L13
Article ID: scipharm.2010.78.553
Subjects: Conference abstract L13

Translational Medicine: Pharmacokinetic and Pharmacogenetic Aspects of Personalized Pharmacotherapy

Grabnar I.
Faculty of Pharmacy, University of Ljubljana, Ljubljana, Slovenia
E-mail: iztok.grabnar@ffa.uni-lj.si
Electronic publication date: 2010 Sep 30
Print publication date: 2010 Jul-Sep
Volume: 78
Issue: 3
First page: 553
Last page: 553
Copyright: © author(s); licensee Österreichische Apotheker-Verlagsgesellschaft m. b. H., Vienna, Austria.
Copyright year: 2010
License: This is an Open Access article distributed under the terms of the Creative Commons Attribution License (http://creativecommons.org/licenses/by/3.0/), which permits unrestricted use, distribution, and reproduction in any medium, provided the original work is properly cited.

JOURNAL INFORMATION
==============================
NLM Title Abbreviation: Sci Pharm
Journal ID: Sci Pharm
NLM Journal ID: 0026251
Journal ID: Scientia Pharmaceutica

Scientia Pharmaceutica

ISSN: 0036-8709
EISSN: 2218-0532
Publisher: Österreichische Apotheker-Verlagsgesellschaft, m. b. H.

ARTICLE INFORMATION
==============================
DOI: 10.3797/scipharm.cespt.8.L14
Article ID: scipharm.2010.78.554
Subjects: Conference abstract L14

Geriatric Therapeutic Care – A Story to Tell about my Grand-Parents

Stegemann S.
Capsugel a division of Pfizer, Rijksweg 11, 2880 Bornem, Belgium
E-mail: sven.stegemann@pfizer.com
Electronic publication date: 2010 Sep 30
Print publication date: 2010 Jul-Sep
Volume: 78
Issue: 3
First page: 554
Last page: 554
Copyright: © author(s); licensee Österreichische Apotheker-Verlagsgesellschaft m. b. H., Vienna, Austria.
Copyright year: 2010
License: This is an Open Access article distributed under the terms of the Creative Commons Attribution License (http://creativecommons.org/licenses/by/3.0/), which permits unrestricted use, distribution, and reproduction in any medium, provided the original work is properly cited.

The author would like to thank all members of the ‘Geriatric Medicines Society’ for the contributions to this paper.

This work is supported by the European Research Council through grant number 200580-Chobotix.

This work was supported by the German Research Foundation (DFG SPP 1313), the Swiss National Foundation (Nr. 3100A0_118420), the Doerenkamp Zbinden Foundation and the AnimalFreeResearch.

